# Multi-Phenotypic subtyping of circulating tumor cells using sequential fluorescent quenching and restaining

**DOI:** 10.1038/srep33488

**Published:** 2016-09-20

**Authors:** Daniel L Adams, R. Katherine Alpaugh, Susan Tsai, Cha-Mei Tang, Steingrimur Stefansson

**Affiliations:** 1 Creatv MicroTech, Inc., 1 Deer Park Dr. Monmouth Junction, NJ 08850, USA; 2 Fox Chase Cancer Center, Protocol Support Laboratory, 333 Cottman Ave., Philadelphia, PA 19111, USA; 3 The Medical College of Wisconsin Milwaukee, Milwaukee, WI 53226, USA; 4 Creatv MicroTech, Inc. 9900 Belward Campus Dr., Rockville, MD 20850, USA; 5 HeMemics Biotechnologies Inc., 12111 Parklawn Drive Rockville, MD 20852, USA

## Abstract

In tissue biopsies formalin fixed paraffin embedded cancer blocks are micro-sectioned producing multiple semi-identical specimens which are analyzed and subtyped proteomically, and genomically, with numerous biomarkers. In blood based biopsies (BBBs), blood is purified for circulating tumor cells (CTCs) and clinical utility is typically limited to cell enumeration, as only 2–3 positive fluorescent markers and 1 negative marker can be used. As such, increasing the number of subtyping biomarkers on each individual CTC could dramatically enhance the clinical utility of BBBs, allowing in depth interrogation of clinically relevant CTCs. We describe a simple and inexpensive method for quenching the specific fluors of fluorescently stained CTCs followed by sequential restaining with additional biomarkers. As proof of principle a CTC panel, immunosuppression panel and stem cell panel were used to sequentially subtype individual fluorescently stained patient CTCs, suggesting a simple and universal technique to analyze multiple clinically applicable immunomarkers from BBBs.

Circulating Tumor Cells (CTCs) are cancer cells that shed from primary/metastatic solid tumors and can be found transiting the circulatory system^1^,^2^,^3^,^4^,^5^,^6^,^7^,^8^,^9^,^10^,^11^,^12^,^13^,^14^,^15^. For many years whole peripheral blood has been used to isolate CTCs from cancer patients for use as a prognostic indicator of advanced disease^1^,^2^,^4^,^5^,^7^,^9^,^10^,^12^,^14^,^15^,^16^,^17^. Currently, the only clinically validated prognostic assay isolates CTCs based on antibody mediated capture and identifies CTCs based on 3 cellular fluorescent markers^1^,^2^,^3^,^4^,^5^,^6^,^7^,^8^,^9^,^10^,^11^,^12^,^14^. This FDA approved assay (CellSearch^®^ CTC Test) captures CTCs from blood using ferrofluid nanoparticles conjugated with antibodies against the epithelial cell adhesion molecule (EpCAM). Then, like most CTC systems, captured cells are identified using 3 fluorescent markers, DAPI (to stain nuclei and identify an object as a cell), cytokeratin (CK) (to identify the cell as epithelial), and CD45 (to exclude white blood cells)^1^,^2^,^3^,^4^,^5^,^6^,^7^,^8^,^9^,^10^,^11^,^12^,^14^.

Recently a plethora of alternative CTC isolation methods have been introduced, many of which attempt to expand the clinical utility of CTCs beyond simple enumeration^1^,^2^,^3^,^4^,^9^,^11^,^12^,^13^,^14^,^18^. However, irregardless of the isolation platform fluorescence detection is usually the identification criteria used, and remains mostly limited to 4–5 total fluors^1^,^2^,^3^,^4^,^7^,^8^,^9^,^11^,^12^,^14^,^15^,^16^,^18^,^19^. This limits fluorescence based CTC characterization to the 3 fore mentioned identification biomarkers and 1–2 additional subtyping biomarkers^20^,^21^,^22^,^23^,^24^. Clinically and biologically this limits researchers to superficial proteomic identification of CTCs, while the need to truly interrogate relevant tumor cell phenotypes requires multiple subtyping markers^1^,^2^,^3^,^4^,^9^,^10^,^11^,^14^,^15^,^16^,^17^,^18^,^19^,^25^,^26^,^27^,^28^,^29^,^30^. While it is possible to partially overcome this limitation by isolating cells using multiple blood samples from the same patient^1^,^3^,^4^,^10^,^13^,^14^, typically less than 50 mL is allowed to be drawn. Further, CTCs have enormous phenotypic heterogeneity which makes the staining of individual CTCs from different blood draws incomparable^1^,^2^,^3^,^4^,^9^,^12^,^14^,^15^,^16^. If, like in sectional tissue biopsies, a method existed to analyze CTCs using multiple markers against each isolated cell, the ability to biologically interrogate individual cells would greatly enhance the clinical utility.

Identification of CTCs is complicated, as different subgroups of cancer cells upregulate and/or down regulate phenotypes in relation to tumor progression, tumor spread, and in response to tumor treatments^1^,^2^,^3^,^4^,^9^,^11^,^12^,^14^,^15^,^16^,^17^,^29^,^30^,^31^. The ability of individual cancer cells to transition states, such as the epithelial to mesenchymal transitions (EMT), or alter expression of inflammatory immune checkpoints are examples of the active state of tumors changing dynamically in real time as the cancer progresses or responds to treatment. As such, CTCs are uniquely suitable as a possible representative surrogate biomarker for tracking a tumor’s transient states in real time^1^,^2^,^3^,^4^,^9^,^11^,^12^,^14^,^15^,^16^,^17^,^29^.

CTCs undergoing EMT are common constituents in cancer patient blood, which have been implicated as a primary cellular component in metastatic spread^2^,^4^,^9^,^15^,^16^,^18^,^19^,^28^,^29^,^30^,^31^. Unfortunately, EMT has no universally accepted positive set of biomarkers and is generally described by the down regulation of epithelial proteins, e.g. EpCAM and CK, and the upregulation of mesenchymal stem cell proteins, e.g. vimentin and CD34^2^,^4^,^9^,^14^,^15^,^16^,^18^,^19^,^27^,^28^,^29^,^30^,^31^,^32^,^33^. EMT is currently a topic of great interest^2^,^3^,^4^,^9^,^11^,^14^,^15^,^16^,^19^,^27^,^28^,^29^,^30^,^31^,^32^,^33^,^34^, however, because of the limited proteomic analysis from the limited free fluorescent channels, EMT subtyping is typically screened using non-proteomic methods, i.e. mRNA expression or DNA analysis^2^,^3^,^4^,^9^,^11^,^14^,^15^,^16^,^19^,^27^,^28^,^29^,^30^,^31^,^32^,^33^,^34^.

In fluorescence based staining of biological samples, borohydride derivatives (i.e. Cyanoborohydride and Lithium Borohydride) are staple reagents used to reduce background autofluorescence without harming proteomic/genomic markers. Interestingly, while borohydride derivatives are used to quench nonspecific fluorescence in biopsied micro-sections, it is not generally used to remove specific fluorescence^20^,^21^,^22^,^23^,^24^,^33^,^35^,^36^,^37^. Borohydride (BH_4_) is a mild and selective carbonyl reducing agent that is commonly used in organic chemistry to reduce ketones and aldehydes to alcohols, and/or imines to secondary amines, without reducing amide or carboxy acid functional groups^20^,^21^,^22^,^23^,^24^,^33^,^35^,^36^,^37^. In microscopy, BH_4_ derivatives are often used to quench the autofluorescence of formaldehyde, and glutaraldehyde, fixed biological samples which is caused by accumulation of carbonylated and Schiff-base compounds^38^. A secondary benefit of adding BH_4_ derivatives to fixed biological samples is the ability to reduce free aldehyde groups (e.g. aldehyde blocking), which further minimizes nonspecific binding of the histochemical reagents. In microscopy, samples are quenched with BH_4_ derivatives before staining with fluorescent biomarkers, greatly reducing non-specific background fluorescence without damaging the protein epitopes^20^,^21^,^22^,^23^,^24^,^35^,^36^,^37^,^38^.

An advantage of a filter based platform is that each individual cell is fixed *in situ*, imaged, positioned, marked and archived^1^,^2^,^3^,^25^,^39^, allowing the user to relocate the identical cell after each step in the quenching process. We initially screened and optimized a process of quenching, underivatizing, amine stripping, and restaining (QUAS-R) on model cell lines which had been isolated on a CellSieve™ microfiltration system^1^,^2^,^3^,^25^,^39^. After verifying epitope stability in cell lines during the quenching process, we then tested the process on 12 previously screened and archived pancreatic cancer patient samples. As a proof of principle using previously identified EMTCTCs^1^,^2^,^39^, we were able to rapidly screen these cells for mesenchymal stem cell markers (CD34 and Vimentin)^27^,^34^, motility markers (CXCR4 and Vimentin)^27^,^28^,^30^,^34^, and inflammatory markers (PD-L1 and PD1)^40^ to characterize the expression profiles and typing of EMTCTCs found in pancreatic cancer patients. Our data suggest that sequential fluorescent staining of pre-identified CTCs is possible, the protein epitopes appear stable, and the markers can be visualized for accurate cytological assessment, much like classical cancer histopathological subtyping. In total, we describe the ability to sequentially analyze, subtype and track 9 distinct phenotypic cancer markers on every single isolated EMTCTC.

## Results

### Model Cell Lines

**Figure 1:**
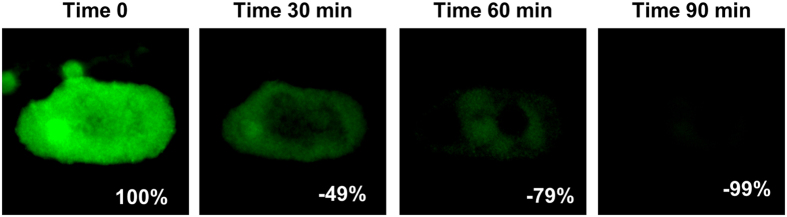
Tracking the quench time of borohydride solution in the removal of visible and quantifiable fluorescence from patient blood cells. A Cytokeratin positive cell isolated from a pancreatic cancer patient was identified, imaged and the signal measured at time 0. Borohydride solution was added and the cell was imaged 30 min, 60 min and 90 after induction of borohydride. After 90 min, 99% of the original fluorescence had been quenched. Box scale = 45 μm.

We first tested the ability of our method to quench fluorescence using the MDA-MB-231 cell line labeled with fluorescent CTC markers (Supplementary Figures 1 and 2). After incubation with a borohydride solution for 1.5 hours, the FITC fluorescent signal on the MDA-MB-231 cells visually disappeared (Supplementary Figures 1 and 2). We then confirmed this quenching technique on FITC labeled patient sample cells by imaging cells stained with cytokeratin tagged FITC, after 30, 60 and 90 minutes of incubation in borohydride (Fig. 1). We calculated the quenching by measuring the fluorescent intensity of the cells at the 4 time points and found that 99% of the cellular fluorescence was quenched after 90 min of incubation (Fig. 1).

**Figure 2:**
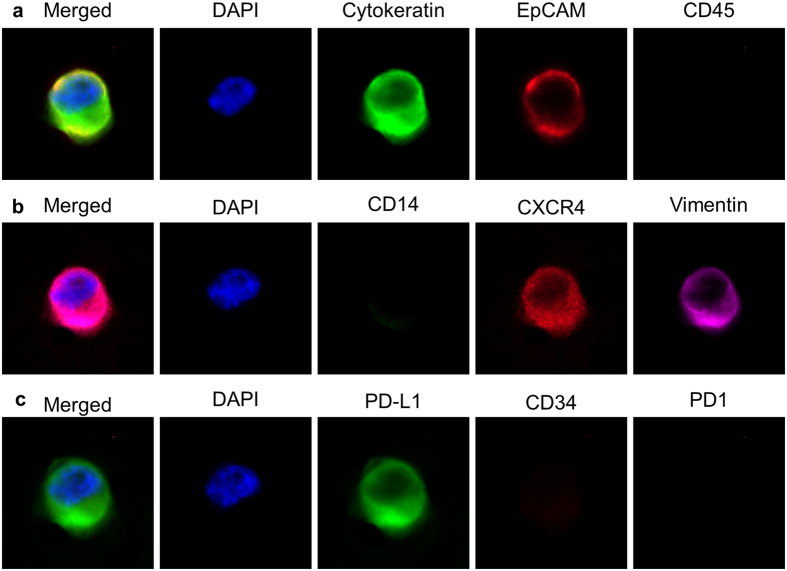
Visual example of a MDA-MB-231 cell through the 2 QUAS-R rounds. **(a)** After filtration, a MDA-MB-231 cell was stained with the CTC stain Cytokeratin, EpCAM and CD45. (**b)** The cell was quenched by QUAS-R and stained with CD14, CXCR4 and Vimentin. (**c**) The cell was quenched again by QUAS-R and stained with PD-L1, CD34, and PD1. Box scale = 45.

**Figure 3:**
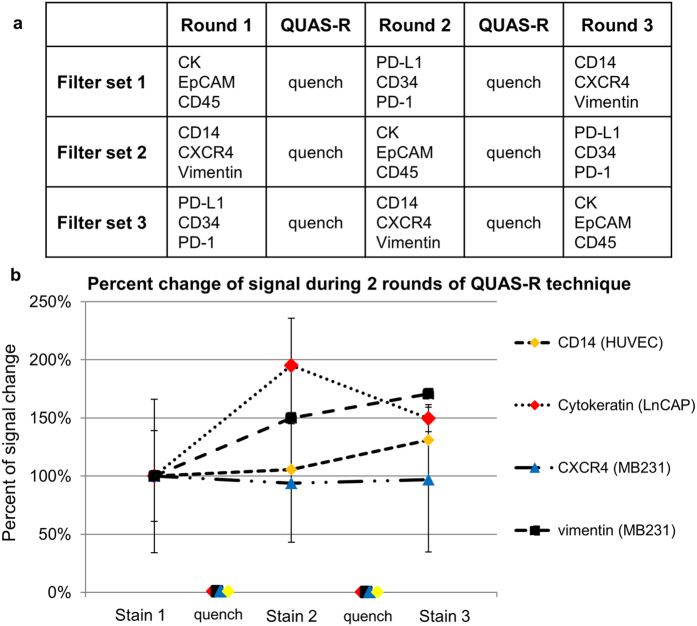
Overview of experimental design and representative examples of the percent change of signal intensity when a marker stain was used on the first round, second round or third round of staining. (**a**) Stains were tested on 5 cell lines at three separate time points. A 1^st^ set of stains were used followed by quenching. After quench, a 2^nd^ set of stains were used followed by quenching. After quenching, a 3^rd^ set of stains were used. After each round, cells were imaged and quantified. b. Representative of signal intensities shows no degradation irregardless of whether the stains were used first, second or third. A repeated measure ANOVA found no significant difference in signal intensity between the 3 staining times [vimentin MDA-MB-231 (p = 0.201), cytokeratin LNCaP (p = 0.291), CD14 HUVEC (p = 0.499), and CXCR4 MDA-MB-231 (p = 0.857)]. Full comparison of all cells and all markers is found in Supplementary Figures 2 and 10.

Optimally, blood based biopsies (BBBs) would analyze multiple types of circulating cells (i.e. CTCs, Circulating endothelial cells, etc.) with many different markers. However, to rescreen samples the stability of cellular epitopes must remain consistent in between assays. Therefore, we tested QUAS-R on 5 distinct model cell lines (MDA-MB-231, MCF-7, LnCAP, A2058, and HUVEC) of breast, endothelial, prostate and melanoma origin with respect to epitope stability (Figs 2, 3 and Supplementary Figure 3). Since Immunohistochemistry (IHC) is used in cancer subtyping for both intracellular and extracellular epitopes, we specifically chose 9 markers with a broad range of cellular localization, which occur intracellularly (cytokeratin and vimentin) and extracellularly (EpCAM, CD45, CD31, CD34, PD-L1, CXCR4 and CD14), Fig. 2 and Supplementary Figure 3. A baseline signal for all of the 9 markers, with all 5 cell lines on separate filters was performed. One filter set, each with one of the 5 cell lines (A2058, LnCAP, MDA-MB-231, MCF-7 or HUVECs) was stained using the CTC markers (CK-FITC, EpCAM-PE and CD45-Cy5)^1^,^2^,^3^,^4^,^5^,^7^,^8^,^9^,^11^,^12^,^13^,^14^,^15^,^16^,^17^,^19^,^26^,^27^,^29^,^30^,^32^,^39^. On a second set of filters, the 5 cell lines were stained with a panel of PD-L1-FITC, CD34-PE and PD-1-Dylight 650. On a third set of filters, the 5 cell lines were stained with a panel of CD14-FITC, CXCR4-PE and vimentin-efluor660 (Fig. 3a). We measured the intensity of each marker (10 cells for each marker) and normalized it to background, for ease of comparison (Fig. 3 and Supplementary Figures 2 and 3). We then performed QUAS-R on all filters and restained each set with a second marker panel (Fig. 3a). We again measured the intensity of each marker for all 5 cell types and normalized to background (Supplementary Figure 3). We performed a second QUAS-R and restained each filter set with a third marker panel (Fig. 3a). Visually, and quantifiably, CD45 was correctly negative in all cells, Fig. 2 and Supplementary Figure 3. CD14 and CD34 were weakly positive in the HUVEC cells (Supplementary Figure 3), caused by the high variable nature of HUVECs. Further, the CD14 signal was localized to the peripheral protrusions of a subset of HUVECs (Supplementary Figure 4) causing the interior of the cell to have low/no signal. EpCAM also appeared low in expression and high standard deviation (STD), as the signal was highly heterogenous between cells, and the receptors were aggregated on the cell surface, causing cells to have areas of high and low signals (Supplementary Figure 5). CD184 was correctly localized to the MDA-MB-231 cell lines, albeit with a large STD from the highly varying cell populations (Fig. 2 and Supplementary Figure 3). PD-L1 and vimentin all showed intense staining in the correct cell lines (MDA-MB-231:high vimentin/high PD-L1, A2058:high vimentin/high PD-L1, and HUVEC:high vimentin) and low/no staining in the correct cell lines (HUVEC:no PD-L1, LNCaP:no vimentin/low PD-L1, and MCF-7:no vimentin/low PD-L1), while Cytokeratin stained MCF-7 MDA-MB-231 and LNCaP strongly. Importantly, none of the biomarkers diminished in intensity between the two restains and appeared with appropriate staining intensity in each of the proper cell lines (Supplementary Figures 3, 6–9). The possible exception was PD-L1, which did appear to slope down during the 3 stain, but was within the STD. Taken together, these experiments suggests that QUAS-R allows for the full quenching of fluorescently labeled cells and restaining biological specimens, without negatively affecting the quality of their epitopes.

### Patient Samples

It has been shown that a standard CellSieve™ CTC fluorescent staining panel can identify, quantify and score the same clinically prognostic CTCs as the FDA approved CellSearch^®^ System^1^,^2^. However, we also identified numerous subtypes of CTCs with EMT-like phenotypes, including cells with downregulated cytokeratin and loss of EpCAM^1^,^2^,^39^. While the downregulation of epithelial markers is a hallmark of EMT in cancer, additional confirmation of upregulated mesenchymal markers is needed to properly profile their stem cell and motility characteristics.

**Figure 4:**
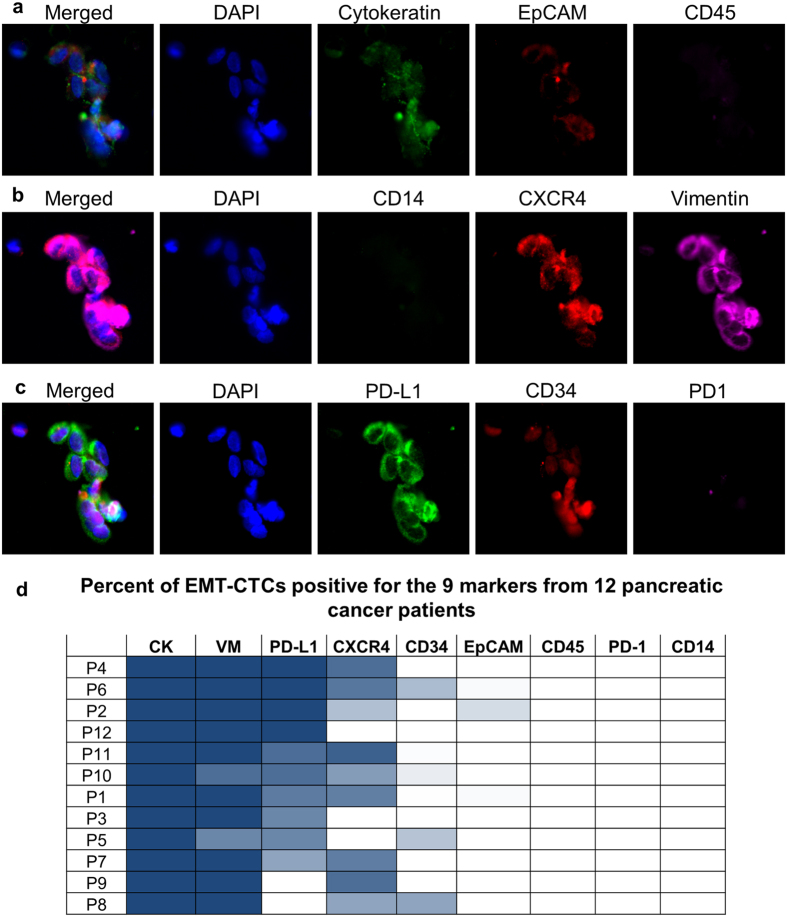
QUAS-R on patient derived EMT-like CTCs for subtyping cells and screening drug targets. **(a)** A proof of principle study of 12 pancreatic cancer patient samples were imaged with the CTC stains CK, EpCAM and CD45. EMT-Like CTCs were enumerated and imaged. (**b**) QUAS-R was performed and cells were stained with the subtyping markers CD14, CXCR4, and Vimentin. (**c)** QUAS-R was then performed again and cells stained for the drug targets PD-L1, CD34 and PD1. (**d**) A total of 764 EMTCTCs with a median of 10 cells per sample were measured for presence of the 9 markers. Presence of each marker in each patient is represented as a heat map of percent EMT-CTCs positive for each stain. VM = vimentin, CK = cytokeratin, numerical values can be found in Supplementary Figure 11. Box scale = 75 μm.

To better study EMTCTCs we used pancreatic cancer patient samples that had been previously enumerated and imaged using the CTC panel as containing EMTCTCs^1^,^2^,^39^. Specifically, EMTCTCs were found in 78% of pancreatic samples, irregardless of stage^39^, while none of the 12 healthy control samples had EMTCTCs, CAMLs, or PDCTCs. In the set of 12 patient samples there were a total of 764 EMTCTCs with a median of 10 cells per sample. A representative image of pancreatic EMTCTCs, from a stage IV pancreatic sample, with the expression profiles for CK, EpCAM and CD45 is shown in Fig. 4a. QUAS-R was performed on all 12 patient samples. All cells were then restained and reimaged with anti-CD14, anti-CXCR4 and anti-vimentin, Fig. 4b. After imaging all cells, QUAS-R was performed a second time on each patient sample and then restained with anti-PDL-1, anti-CD34 and anti-PD-1 (Fig. 4c). Each EMTCTC was counted and the expression phenotypes were quantified, Fig. 4d.

Not surprisingly, all EMTCTCs were positive for CK and negative for CD45, both being the markers used to identify CTCs. CD14 and PD-1 were also negative in all EMTCTCs, as these are markers for macrophages and activated T-cells, respectively. EpCAM was very uncommon in this cell type, occurring in only 2% of the cell population (Fig. 4d). The absence of EpCAM in EMTCTCs is a well-recognized as part of the EMT process and in agreement with previous reports^2^,^4^,^9^,^11^,^14^,^15^,^16^,^19^,^27^,^29^,^30^,^31^,^39^. Vimentin was the next most prevalent marker found in all but 3 cells, or 99% of all cells. This is not surprising as the EMT process is reported to downregulate cytokeratin and upregulate vimentin^9^,^27^,^29^,^30^,^31^. This process has been shown to increase a cell’s mesenchymal phenotypes and allow cells improved motility^9^,^27^,^29^,^30^,^31^. Interestingly the prevalence of CXCR4 in some patients indicates that these mesenchymal cells are likely migratory^27^,^28^,^34^. Expression of PD-L1 and CXCR4 was highly heterogeneous between patients, ranging from 100% to 0% positivity for PD-L1 and 90% to 0% positivity for CXCR4. Additionally CD34 was rarely found, with only 5 cells in 3 patients being weakly positive, but since this marker is associated with progenitor stem cells and/or endothelial progenitor cells, further evaluation of endothelial subtyping and patient outcome will be of interest^33^.

Using a standard CTC fluorescent stain, we identified, quantified and scored EMTCTCs according to their cytokeratin, EpCAM and CD45 presence^1^,^2^,^3^,^39^. We then specifically screened these EMTCTCS to better understand and interrogate their underlying subtypes. This study indicates that circulating cells from filtered patient samples can be processed by the QUAS-R technique, whereby a fluorescent CTC stain is quenched while leaving the cells intact and unaffected. We describe that samples can be restained for subtyping with additional biomarker panels against biological targets (i.e. PD-L1, PD-1, etc.). Additionally, during 2 rounds of QUAS-R no degradation was quantified in any of the cell lines in their surface receptors, nor intracellular markers. These experiments suggest that previously identified CTCs can be reimaged and subtyped with multiple additional immuno-targets, with markers quantified and scored much like a multipanel IHC testing in classical formalin fixed paraffin embedded (FFPE) cancer biopsies.

## Conclusion

CTCs are rare but clinically important prognostic indicators of cancer patient status, however, their clinical utility remains limited to their enumeration using 2–3 positive fluorescent markers and 1 negative fluorescent marker. This is in stark contrast to classical tissue biopsies that yield vast clinical information about the underlying tumor biology and in guiding patient treatment. In cancer pathology, IHC is the standard biological assay accomplished using thin slices (~5–10 μm) cut from a large FFPE biopsied tissue section. With multiple slices and each slice with hundreds of tumor cells, a variety of subtyping assays can be run on the semi-identical tumor cells for use in staging, prognosis and in predicting treatment response. Blood based biopsies (BBBs) typically only isolate a few CTCs (0-81 CTCs/7.5 mL sample) and the majority of samples (74%) contain < 2 CTCs^1^,^2^,^3^,^4^,^5^,^7^. If CTC analysis is to live up to its promise as a “blood-based biopsy”, then it must demonstrate the ability to not only identify tumor cell presence, but proteomically subtype the cells for clinically relevant markers.

It has been previously described that CTCs with the EMT phenotype (i.e. low cytokeratin and low/no EpCAM) are commonly isolated from breast, prostate, lung and pancreatic cancers^2^,^39^. After an initial enumeration of EMTCTCs in patients with pancreatic cancer for use in clinical prognostication, further interrogation was not possible as the cell stains had occupied all fluorescent channels (i.e. FITC conjugated CK, PE conjugated EpCAM, DAPI and Cyanine5 conjugated CD45). As multiple biomarker interrogation is necessary to properly identify EMT cells, and there is no single biomarker panel for EMTCTCs, testing these cells with a panel of EMT indicative biomarkers would be optimal for proper analysis. The need to expand the repertoire of CTC biomarker subtyping prompted us to examine quenching procedures that could be used to interrogate each CTC with multiple fluorescent antibodies^1^.

The technical difficulty in profiling CTCs cannot be underestimated since cancer patient blood samples usually contain ~1 CTCs per 10^9^ blood cells, which limits options possible for detailed cancer cell profiling. Complicating the matter is that these cells must be identified fluorescently with DAPI, cytokeratin and CD45; leaving only 1–2 channels available for proteomic subtyping^1^,^2^,^3^,^4^,^5^,^7^,^8^,^10^,^12^,^16^,^17^,^19^. Several groups have reported additional staining of CTCs beyond the standard markers (DAPI, EpCAM, CK and CD45), but these have mostly been limited to 1–2 additional markers^4^,^10^,^12^,^16^,^17^,^19^. Examining CTCs isolated from duplicate or multiple samples from the same patients provides some degree of multiplexing^1^,^4^,^10^,^12^,^14^,^15^,^16^,^17^,^19^, however, this option is not ideal since CTCs are rare, very heterogeneous and CTC populations may not be equally distributed in sequential blood samples. The heterogeneity can be seen in clustering CTCs, which only represented 39% of the CTCs enumerated, on par with a number of studies which evaluated clustering of CTCs in pancreatic cancer^41^,^42^,^43^. Within each patient sample, enormous phenotypic variation was found between the individual CTCs and the CTCs within clusters. In the representative cluster, Fig. 4, the variation in intensity between cells can be visualized in cytokeratin, EpCAM, CXCR4, Vimentin, PD-L1 and CD34. The heterogeneity in CTCs is well documented and further suggests that the epitopes are not altered by the QUAS-R technique. In an attempt to expand proteomic subtyping of CTCs, we describe a simple inexpensive quenching and restaining methodology, QUAS-R, which might be used to subtype the same cells with up to 9 unrelated fluorescent antibodies. Specifically, we describe this method for expanding characterization of CTCs from pancreatic cancer patients with the EMT phenotype. Our data suggests that sequential multi-panel restaining of CTCs using clinically applicable cancer biomarkers might provide a greater amount and broader variety of information using a BBB approach.

Recently, it was shown that partial BH_4_ quenching on photoactivatable fluorophores does not alter the quenched proteins and is compatible with high resolution microscopy^37^. The borohydride attributes were described as well suited for temporary reduction of fluorescent signal from biological samples without concern for destruction of epitopes. Using this study as a foundation, we describe that in addition to short term limited dark state quenching, enhanced use of borohydride can be used as a permanent quencher of specific fluors, also without epitope damage. We describe the total removal of IHC specific fluorescence from bound antibodies without harming the visualization or quantification of IHC epitopes. We tested this hypothesis using 6 separate conjugated fluors (alexafluor488, FITC, efluor 660, PE, APC, and Cyanine5), all of which appear to completely darken using this method. Further, while our study focused on BBBs, this method is applicable and transferrable to any cell based assay with a fixed substrate.

Our data suggests that sequential multi-panel restaining of clinically applicable cancer biomarkers might provide a greater amount and broader variety of information using BBBs. In our CTC analysis, we have shown that EMTCTCs are all CK expressing and mostly vimentin expressing. Of course EMT is a transient process defined by a number molecular and proteomic pathways which have not been completely identified. With the ability to stain multiple cell markers, here we looked to further elucidate the underlying biology of CTCs which have appeared to begin the EMT process, i.e. low/negative CK and EpCAM, while verifying that the cells are not originally of hematopoietic (CD45) or myeloid (CD14) lineage. Of course while this initial pilot study focused on a few EMT markers we can now expand the EMT biomarker panel to include all of the known EMT markers on same CTCs. These panels will likely include N-Cadherin, TWIST, SNAIL, ZEB1, etc^14^,^15^,^28^,^29^,^30^,^31^,^32^,^33^,^34^. Further, while the method here uses a combination of manual identification and AxioVision’s Mark and Find module, this was still somewhat time consuming. Considering the simplicity of the reagents used, it is not unreasonable to assume that in the future, a fully automated system adapting the mark and find software might be developed to minimize many of the manual steps. Much like in these methods, semi-automated mark and find software is currently available in a number of microscopy systems. By employing these systems to re-find previously marked cells, autonomous processing will better streamline the process and allow for universal use of this method. However, automated or manual, the ability to screen CTCs beyond basic identification will greatly enhance the clinical utility of liquid biopsies as patient samples can now be screened for prognostic and predictive biomarkers without the need for excess sampling.

## Methods

Methods and any associated references are available in the online version of the paper.

### Online Methods

#### Healthy and Patient Blood Samples

Twelve whole peripheral blood samples were drawn from patients who were actively undergoing treatment for previously confirmed stage I–IV pancreatic cancer at The Medical College of Wisconsin from 2012 to 2013. Samples were collected in accordance with and approved by the local Institutional Review Board (IRB) at the Medical College of Wisconsin, with the patient signed informed consent. All blood samples were drawn into CellSave preservative tubes™ (~9 mL, Janssen Diagnostics) and shipped to clinical core laboratory for processing. Results and patient identification from institutions were not shared or communicated until completion of study. All patient samples were first labeled with a standard antibody mixture for staining epithelial cells consisting of FITC labeled-anti-Cytokeratin 8, 18, 19; r-Phycoerythrin (PE) labeled anti-EpCAM; and Cyanine5 labeled anti-CD45^1^,^2^,^3^,^25^,^39^.

Anonymized healthy volunteers donated blood samples (n = 12) were procured with written and signed informed consent. IRB was in accordance with and approved by Western Institutional Review Board. Donor blood was procured on a voluntarily basis at a blood collection center with no selection process, outside standard exclusion criteria, i.e. all samples were considered from individuals in normal health. All blood samples were drawn into CellSave preservative tubes™ and shipped to a clinical core laboratory for processing.

#### CTC Staining Procedures Performed on CellSieve™ Filters

Samples are filtered with a CellSieve™ Microfiltration Assay using a low-pressure vacuum system which isolates CTCs based on size exclusion, >7 micron, as previously described^1^,^2^,^3^,^25^,^39^. Cells were stained and identified by fluorescent enumeration using CTC enumeration stains, as previously described. Briefly, a low-pressure system uses a filter holder assembly with CellSieve™ filter attached. Peripheral blood (7.5 mL), is diluted in a prefixation buffer and drawn through the filter. The filter is washed, postfixed and permeabilized. The captured cells are stained with an antibody cocktail consisting of FITC-anti-Cytokeratin 8, 18, 19, PE-anti-EpCAM and Cy5-anti-CD45 for 1 hour and mounted with Fluoromount-G/DAPI (Southern Biotech). An Olympus BX54WI Fluorescent microscope with Carl Zeiss AxioCam was used to image the samples. Exposures were preset as 2 sec (Cyanine5), 2 sec (PE), 100–750 msec (FITC), and 10–50 msec (DAPI) for equal signal comparisons between cells. A Zen2011 Blue (Carl Zeiss) with AxioVision Mark and Find module was used to process the images, mark the x/y placement of the cells, and relocate previously imaged cells in a semi-automated manner. Samples were archived and placed in storage at 4 °C for 1 week-2 years.

#### Cell Lines

MCF-7 (HTB-22) and MDA-MB-231 (HTB-26) human breast cancer cell lines; LNCaP (CRL-1740, clone FGC) prostate adenocarcinoma; A2058 (CRL-11147) human skin melanoma cell line; and HUVEC-C (CRL-1730) endothelial cells were procured from ATCC (Manassas, VA). All cell lines were grown in their cell line specified media containing fetal bovine serum (FBS) as recommended by ATCC. Cell lines were maintained in T-75 flasks using prescribed cell culture conditions (5% CO_2_, 37 °C) with media changes every 3–4 days, with the exception of the MDA-MB-231 cell lines, which were grown at 37 °C with no added CO_2_. Cells were harvested using trypsin-EDTA (ATCC Manassas, VA), spun at 125 × g for 5 min resuspended in PBS containing 1% paraformaldehyde. After incubation, cells were diluted in 10x volume of PBS, centrifuged and resuspended in fresh PBS before being spiked into normal blood and isolated using microfilters within 5 min.

#### Additional antibody panels

All antibodies were incubated using the manufacturers’ recommendation of 1 hour at room temp. Primary CTC panel: FITC labeled-anti-Cytokeratin 8, 18, 19; r-Phycoerythrin (PE) labeled anti-EpCAM; and Cyanine5 labeled anti-CD45 (Creatv MicroTech)^1^,^2^,^3^; second panel: Alexafluor 488 labeled anti-PDL1 (2.5 ug/mL) and Dylight 650 labeled PD-1 (5 ug/mL) both PD-L1 and PD-1 were gifts from Dr. Steven Lin, MD Anderson Cancer Center, PE labeled CD34 (2.5 ug/mL, clone 4H11) and third panel: FITC labeled anti-CD14 (5 ug/mL, clone 61D3), PE labeled anti-CD184 (5 ug/mL, clone 2B11), efluor660 labeled anti-Vimentin (2.5 ug/mL, clone V9).

An Olympus BX54WI Fluorescent microscope with Carl Zeiss AxioCam was used to image the samples. Exposures were preset as 2 sec (Cyanine5 and APC), 2 sec (PE), 1000 msec (FITC and Alexafluor 488), 500 ms (efluor660), and 10–50 msec (DAPI) for equal signal comparisons between cells. A Zen2011 Blue (Carl Zeiss) was used to process the images, mark the x/y placement of the cells and relocate previously imaged cells.

#### Fluorescent Quenching (QUAS-R)

Archived samples were removed from storage 1 week to 2 years after initial CTC staining. Samples had been previously imaged and marked prior to subjection to quenching procedures. Slides were soaked in 100 mL PBS for 15 minutes and carefully demounted^25^. Filters were placed into a reaction chamber (Corning) and washed 5 times with 1 mL 1X PBS. Filters with bound cells were incubated with 1 mg/mL borohydride solution (Fisher Scientific) for 1 hour at room temp in a chemical hood. The borohydride solution was removed and filters were washed 6 times with 1 ml PBS. Filters were placed in a clean reaction chamber (Corning) and incubated with 100 mM Tris pH = 9.0 for 1 hour at room temp. Tris was removed and filters were washed 3 times with 1 ml PBS, then 1XPBS/20%FBS was then added to the chamber to block the sample for 30 minutes. After incubation, PBS/FBS solution was removed and the second set of antibody stain was added to the chamber for 1 hour at room temp. After antibody incubation, filters are washed in 1XPBST and slide mounted with Fluoromount-G/DAPI (Southern Biotech). Samples were oriented along the x/y axis and previously imaged cells were relocated using a Zen2011 Blue (Carl Zeiss) software. Images and exposures were preset as above and a Zen2011 Blue (Carl Zeiss) was used to process the images. After imaging the antibody the procedure was repeated with the next antibody cocktail and reimaged as above. For time gated experiments involving visualizing fluorescence quenching, filters were placed under a fluorescent microscope (Olympus) in a ventilated hood and imaged with the filter remaining in the borohydride solution.

#### Sequentially screening biomarkers in model cell lines

Each cell line was individually filtered onto a microfilter and each cell type (n = 3) was stained with either antibody panel 1 (CK, EpCAM, and CD45), antibody panel 2 (PD-L1, CD34, and PD-1), or antibody panel 3 (CD14, CXCR4 and Vimentin), Fig. 3a. After imaging and marking, each individual filter was quenched by the QUAS-R method, as above, and then restained with a second antibody set, i.e. filter set 1 originally stained with CK, EpCAM and CD45 was then stained with antibody panel 2 (PD-L1, CD34, and PD-1); filter set 2 originally stained with PD-L1, CD34, and PD-1 was then stained with antibody panel 3 (CD14, CXCR4, Vimentin); and filter set 3 originally stained with CD14, CXCR4, and Vimentin was then stained with antibody panel 2 (CK, EpCAM and CD45). All originally marked cells were found and reimaged.

After imaging all cell lines, QUAS-R was performed a second time on each filter set and cell line. This time filter set 1 was restained with antibody panel 3, filter set 2 was restained with antibody panel 1, and filter set 3 was restained with antibody panel 1. Again all originally marked cells were found and reimaged.

## Additional Information

**How to cite this article**: Adams, D. L. *et al.* Multi-Phenotypic subtyping of circulating tumor cells using sequential fluorescent quenching and restaining. *Sci. Rep.* **6**, 33488; doi: 10.1038/srep33488 (2016).

## Supplementary Material

Supplementary Information
