# Supplementary material for: Multi-Phenotypic subtyping of circulating tumor cells using sequential fluorescent quenching and restaining
Source: Sci Rep. 2016 Sep 20;6:33488. doi: 10.1038/srep33488 (PMC5028835; doi:10.1038/srep33488)
Supplement: Supplementary Information [file srep33488-s1.pdf]

Multi-Phenotypic subtyping of circulating tumor cells using sequential fluorescent quenching and restaining

Daniel L Adams[1*], R. Katherine Alpaugh[2], Susan Tsai[3], Cha-Mei Tang[4], Steingrimur Stefansson[5]

[1]Creatv MicroTech, Inc., 1 Deer Park Dr. Monmouth Junction, NJ 08850,
[2]Fox Chase Cancer Center, Protocol Support Laboratory, 333 Cottman Ave., Philadelphia, PA 19111
[3]The Medical College of Wisconsin Milwaukee, Milwaukee, WI 53226
[4]Creatv MicroTech, Inc. 9900 Belward Campus Dr., Rockville, MD 20850,
[5]HeMemics Biotechnologies Inc., 12111 Parklawn Drive Rockville, MD 20852.

*Correspondence should be addressed to DL Adams, email: dan@creatvmicrotech.com.

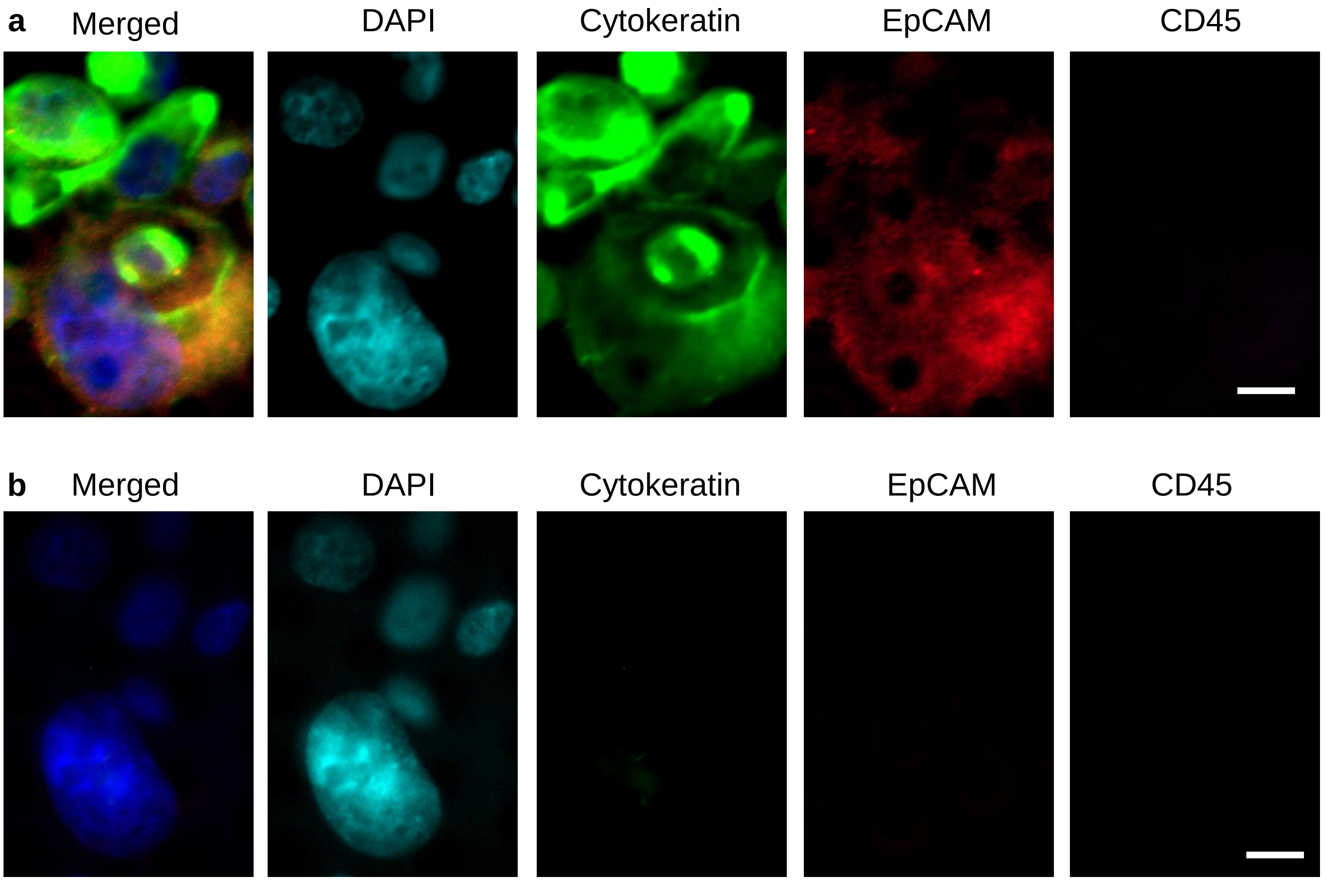

Supplementary Figure 1. Fluorescence quenching of MB231 cell line after 1 hour of borohydride solution. (a) MB231 cell line was filtered using a CellSieve™ filter and stained using the CTC marker panel: DAPI (blue), CK (green), EpCAM (red) and CD45 (violet). (**b)** Filters were quenched for 1 hour, DAPI was added to visualize the cells and the same cells were reimaged using the same exposure times. 100% of the CK signal and 100% of the EpCAM signal was removed. Scale bar=10µm.

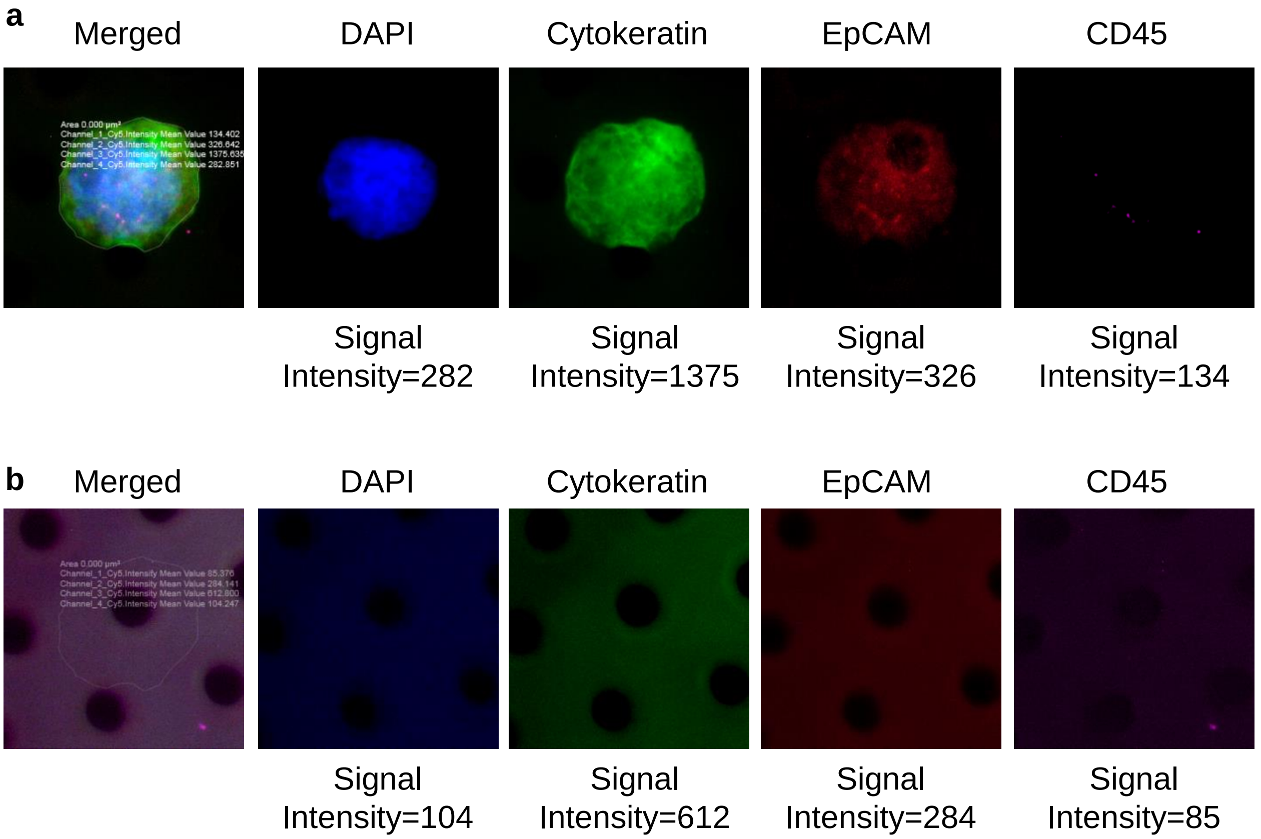

Supplementary Figure 2. **Measuring signal intensity of cells versus background**. (**a**) Each cell is imaged and the average intensity of the signals are measured using Zen2011 Blue, representative MB231 cell line is shown. (**b)** A separate area on the filter, without cells, is also imaged and measured. Subtracting the signal of the cell versus the signal without cells gives the overall stain intensity of each cell.   Box scale=45

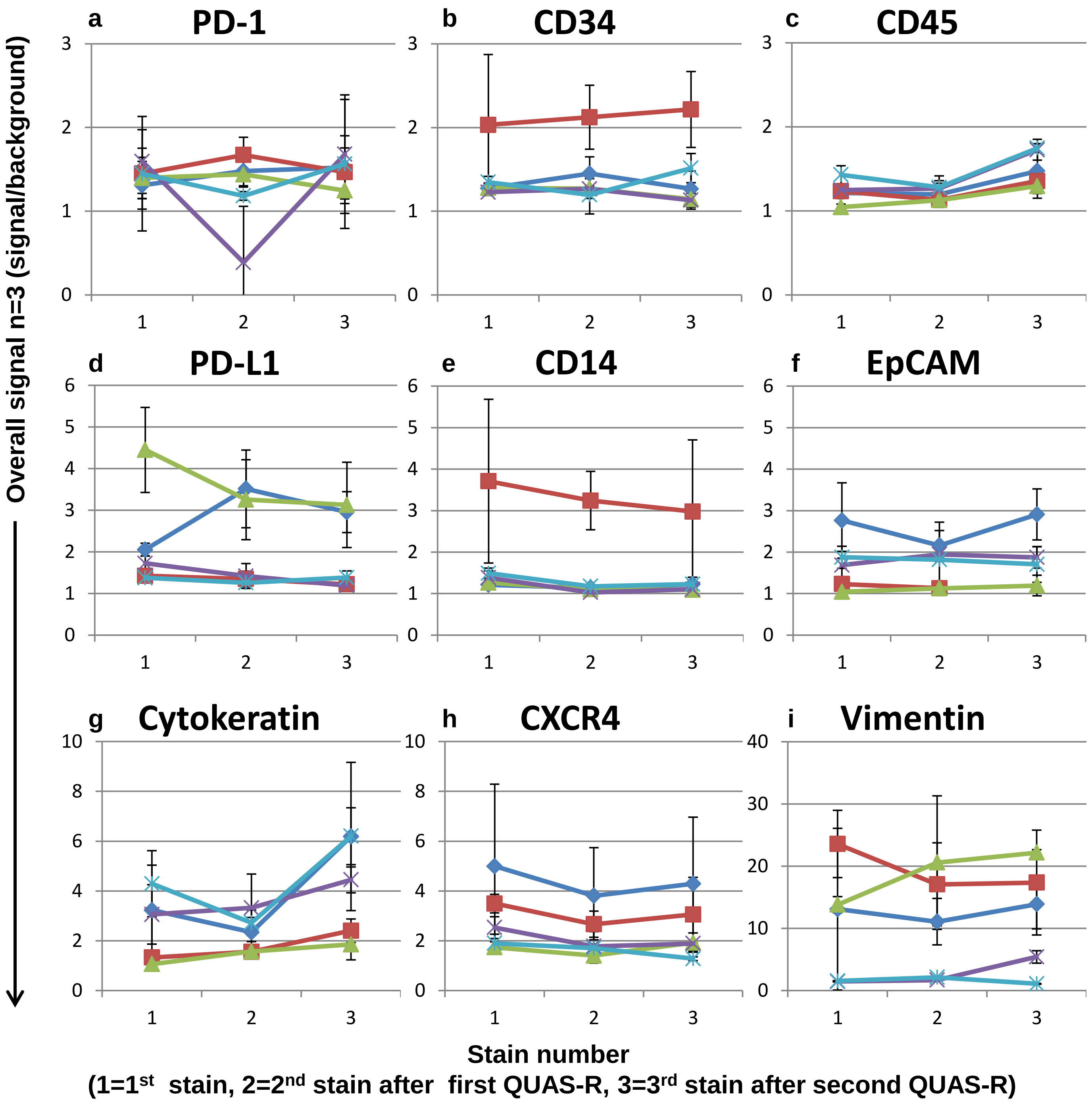

Supplementary Figure 3. Graphing of the overall cellular signal intensity and the changes in 9 cellular markers on 5 separate cell lines through 2 rounds of QUAS-R. (a-i) None of the surface receptors nor intracellular markers degraded in any of the 3 rounds of QUAS-R. **(a)** PD-1 was negative in all the cell lines (**b**) CD34 is weakly positive in the HUVEC cell line and as such appears as a low overall signal. **(c)** CD45 was negative in all the cell lines (**d**) PD-L1 is was variable, seen by the large SD, and largely expressed in A2058 and MB231. (**e**) CD14 was only positive in HUVEC and highly variable as it is only expressed in the protrusions on the cells. (**f**) EpCAM is present as a variable expressing surface marker on LnCAP, MCF-7 and MB231. Low overall signal is caused by localization of the marker (**g**) Cytokeratin is present as intracellular filaments in LnCAP, MCF-7 and MB231. (**h**) CXCR4 is a highly variable surface marker found largely in MB231 and HUVEC cells. (**i**) Vimentin is present as intracellular filament in HUVECs, MB231 and A2058. All signals are normalized to background. The stains are also not effected visually (Figure 2 and Supplementary Figure 1 and 4).

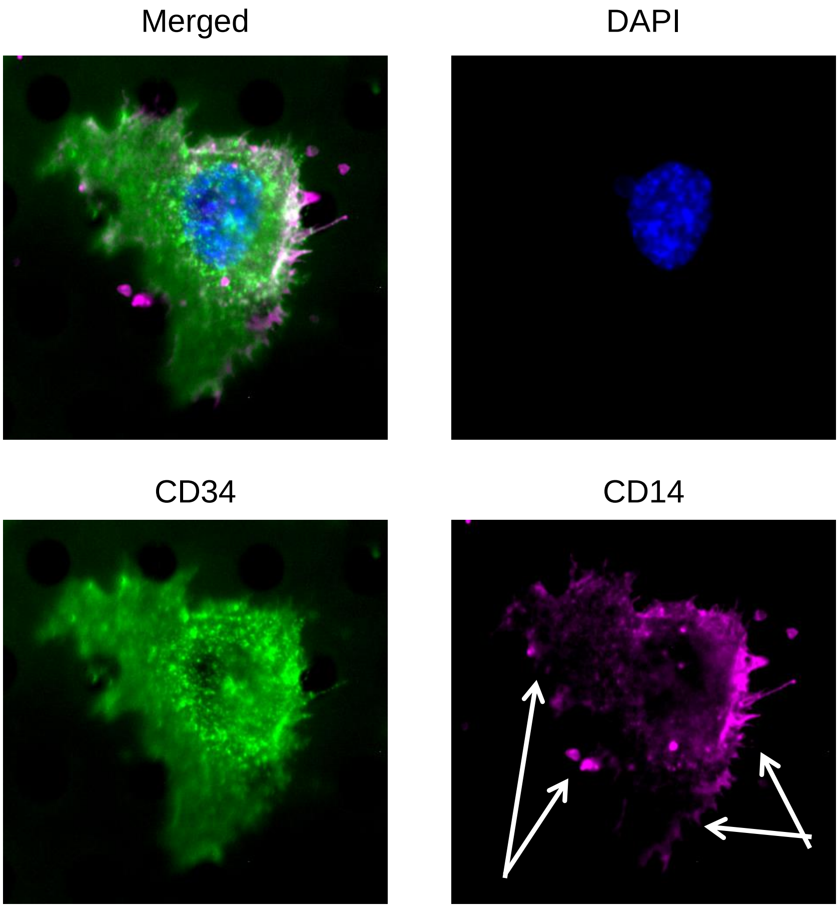

Supplementary Figure 4. HUVEC with DAPI , anti-CD14 and anti-CD34. CD14 is accentuated in the protrusions of the HUVEC cell, while CD34 is found throughout the cell. The intense localized signal of CD14 in only the cell's protrusions results in the cell's total average CD14 intensity to be numerically low despite strong expression .

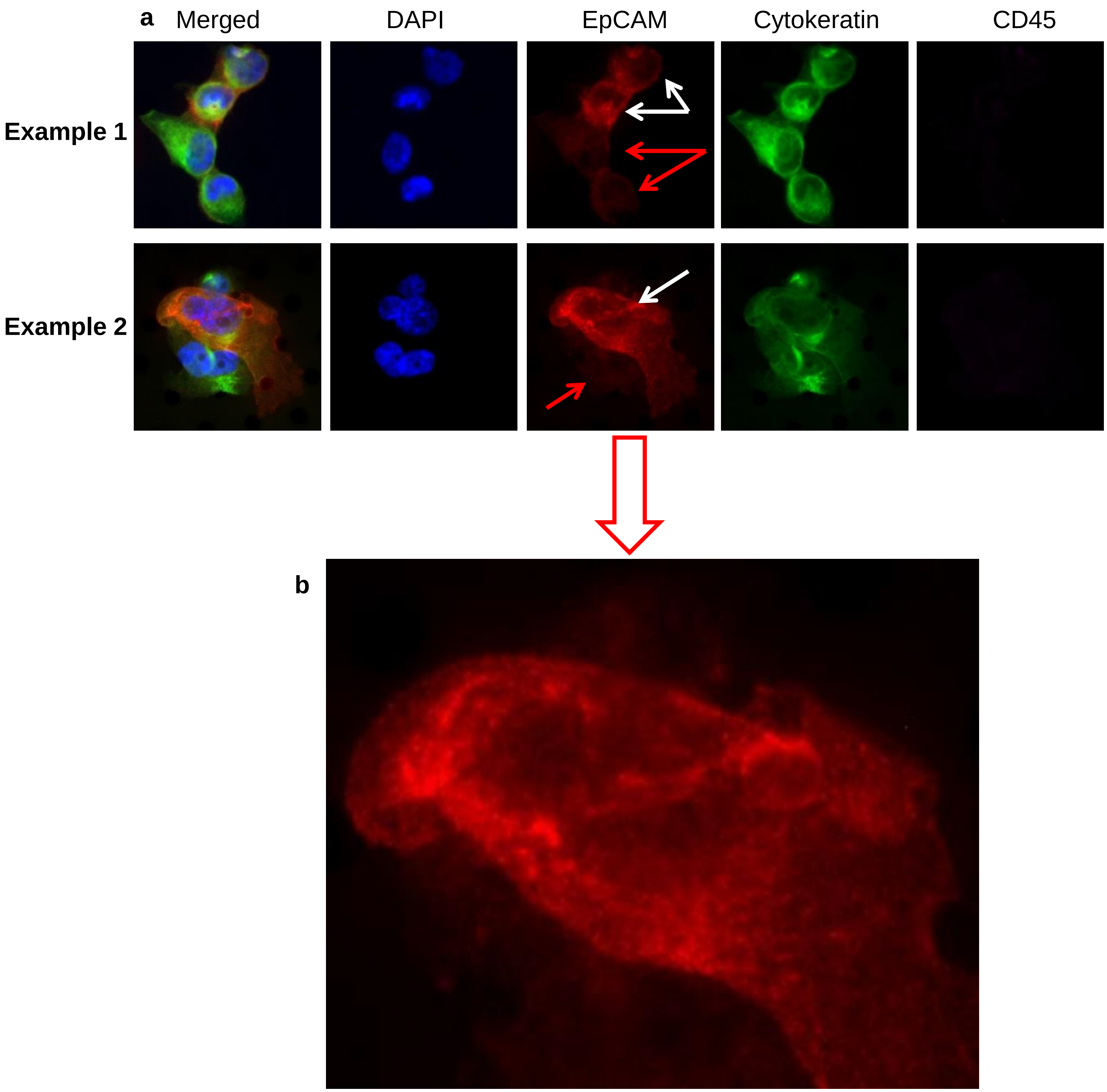

Supplementary Figure 5. A MB231 cultured cell representative of the EpCAM variability and cell surface localization of EpCAM (a) White arrow point to high expressing EpCAM cells while red arrow point to low expressing cells. (**b**) A zoomed image from the EpCAM signal from second panel. EpCAM is diffuse and punctate throughout the cell, causing the overall cell signal intensity to appear numerically low, despite being highly expressed.

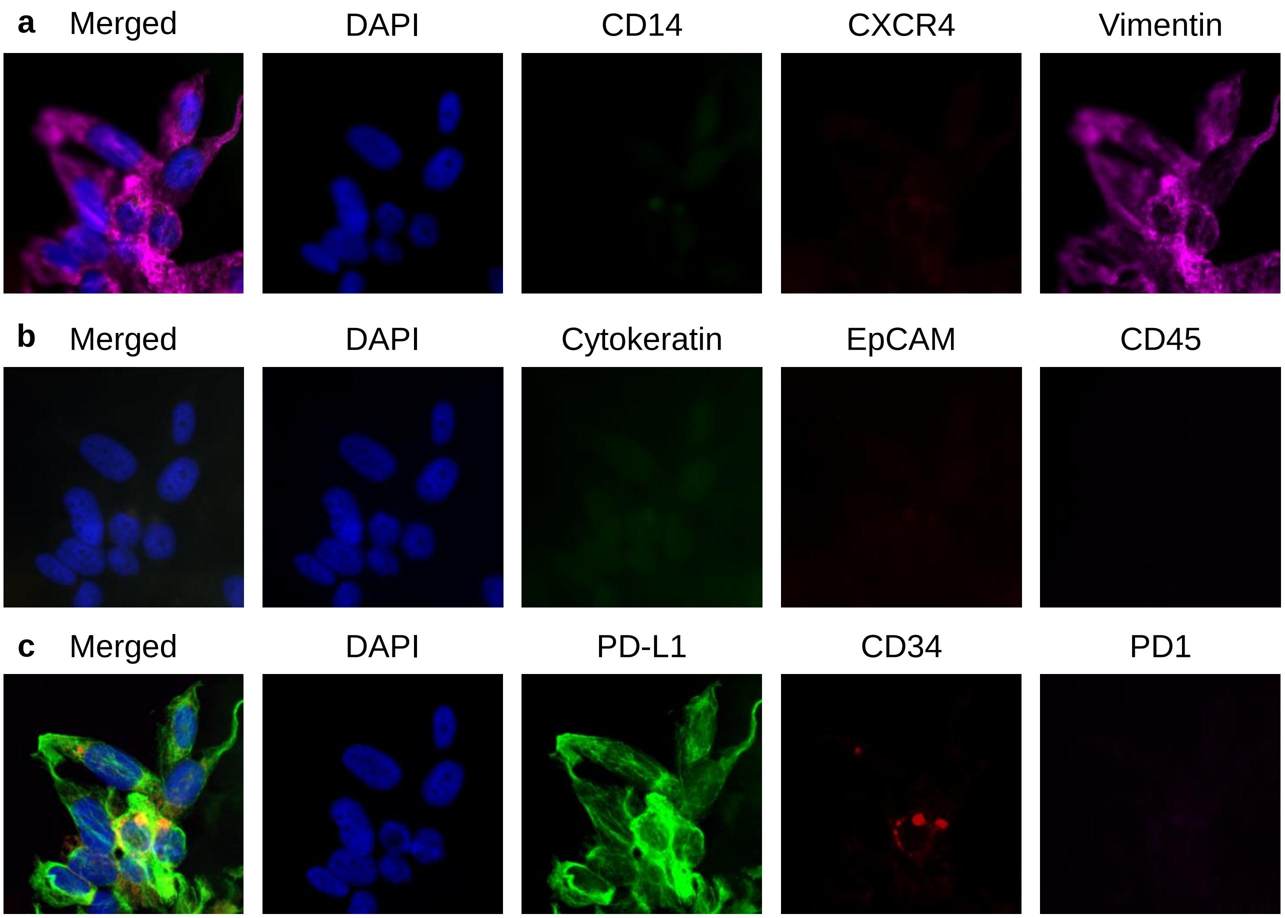

Supplementary Figure 6. Representative example of a A2058 cell line through 2 rounds of QUAS-R in the alternative staining order, compared to Figure 2. a) After filtration, A2058 cells were first stained with CD14, CXCR4 and Vimentin. (**b**) The same A2058 cells were quenched by QUAS-R and stained with Cytokeratin, EpCAM and CD45. (**c**) The same A2058 were quenched again by QUAS-R and stained with PD-L1, CD34, and PD1.

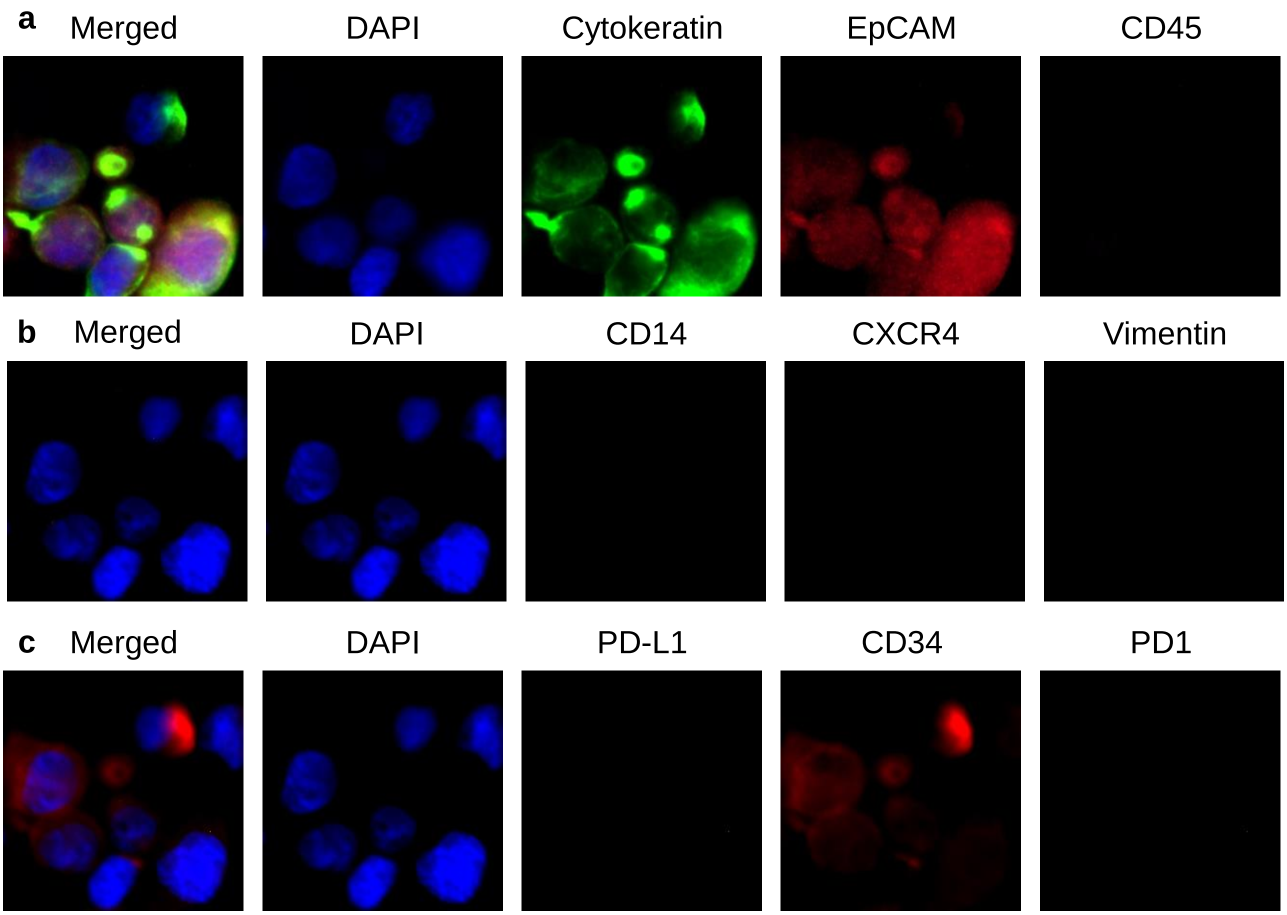

Supplementary Figure 7. Representative example of a LNCaP cell line through 2 rounds of QUAS-R. a) After filtration, LNCaP cells were first stained with Cytokeratin, EpCAM and CD45. (**b**) The same LNCaP cells were quenched by QUAS-R and stained CD14, CXCR4 and Vimentin. (**c**) The same LNCaP cells were quenched again by QUAS-R and stained with PD-L1, CD34, and PD1.

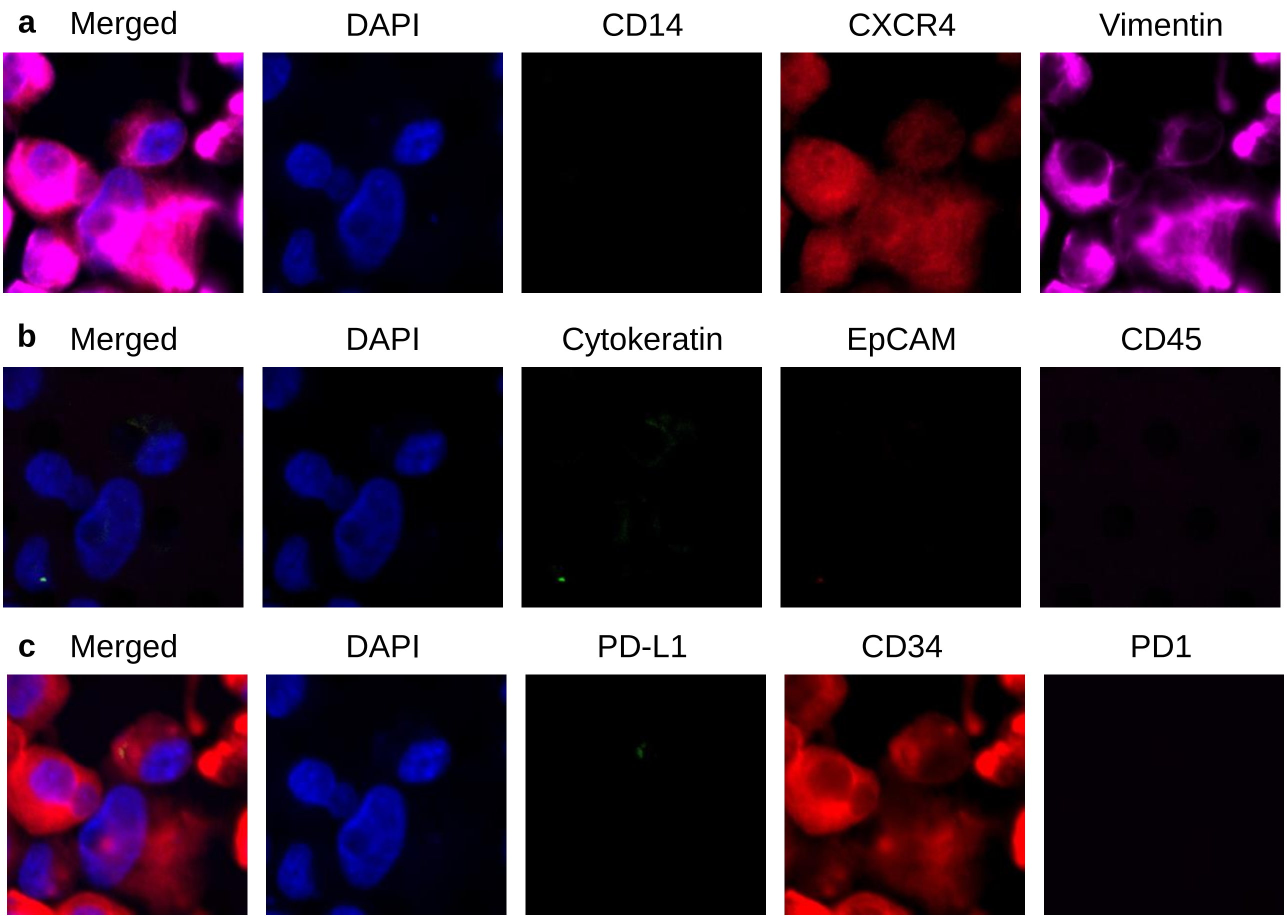

Supplementary Figure 8. Representative example of a HUVEC cell line through 2 rounds of QUAS-R. a) After filtration, HUVEC cells were first stained with CD14, CXCR4 and Vimentin. (**b**) The same HUVEC cells were quenched by QUAS-R and stained with Cytokeratin, EpCAM and CD45. (**c**) The same HUVEC were quenched again by QUAS-R and stained with PD-L1, CD34, and PD1.

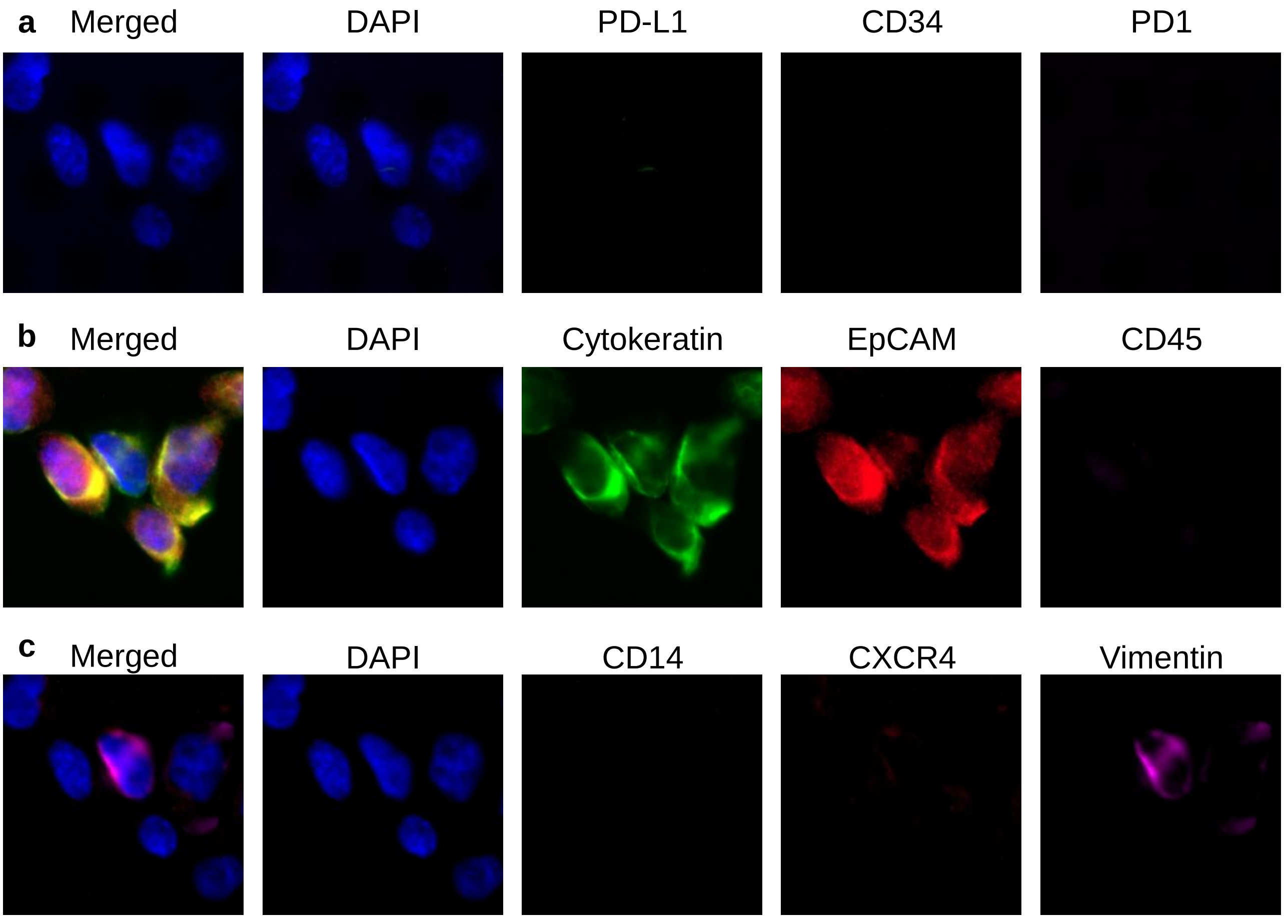

Supplementary Figure 9. Representative example of MCF-7 cell line through 2 rounds of QUAS-R. a) After filtration, MCF-7 cells were first stained with PD-L1, CD34, and PD1. (**b**) The same MCF-7 cells were quenched by QUAS-R and stained with Cytokeratin, EpCAM and CD45. (**c**) The same MCF-7 cells were quenched again by QUAS-R and stained with CD14, CXCR4 and Vimentin.

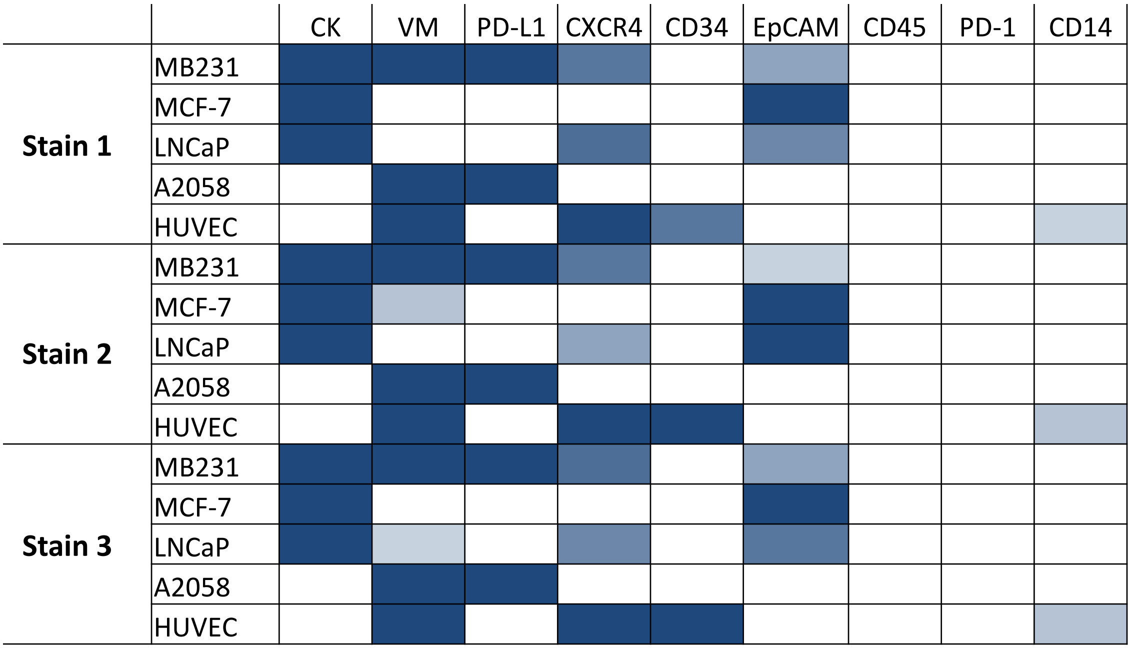

Supplementary Figure 10. Percentages of marker positivity in the 5 model cells line from this study. a) Each marker was measured on each cell line after each step of QUAS-R. Presence of each marker in each cell line for each of the three rounds of staining for each marker which is represented in percent cells positive for each stain. VM=vimentin, CK=cytokeratin.

	CK	VM	PD-L1	CXCR4	CD34	EpCAM	CD45	PD-1	CD14
P4	100%	100%	100%	80%	0%	0%	0%	0%	0%
P6	100%	100%	100%	75%	38%	3%	0%	0%	0%
P2	100%	100%	100%	35%	0%	20%	0%	0%	0%
P12	100%	100%	100%	0%	0%	0%	0%	0%	0%
P11	100%	100%	80%	87%	2%	0%	0%	0%	0%
P10	100%	80%	80%	55%	10%	0%	0%	0%	0%
P1	100%	100%	73%	71%	1%	3%	0%	0%	0%
P3	100%	100%	67%	0%	0%	0%	0%	0%	0%
P5	100%	67%	67%	0%	33%	0%	0%	0%	0%
P7	100%	100%	50%	71%	0%	0%	0%	0%	0%
P9	100%	100%	0%	80%	0%	0%	0%	0%	0%
P8	100%	100%	0%	50%	50%	0%	0%	0%	0%
Average	1	0.956	0.68	0.503	0.111	0.022	0	0	0

Supplementary Figure 11. Raw percentages from Figure b. a) A total of 764 EMTCTCs with a median of 10 cells per sample were measured for presence of the 9 markers. Presence of each marker in each patient is represented in percent EMT-CTCs positive for each stain. VM=vimentin, CK=cytokeratin.
